# Unveiling the enigma: a case of hypercalcemia in end-stage liver disease

**DOI:** 10.17179/excli2025-8663

**Published:** 2025-08-18

**Authors:** Rutvikkumar Jadvani, Abul Hasan Shadali Abdul Khader, Meenu Singh

**Affiliations:** 1 Division of General Internal Medicine, University of Utah School of Medicine, Salt Lake City, Utah, USA

## ⁯

Hypercalcemia is a common clinical problem with a prevalence of 1 in 1000 in the general population. Primary hyperparathyroidism and malignancy account for the majority of cases. Some uncommon under-recognized causes include advanced liver disease and granulomatous diseases (Tonon et al., 2022[9]; Motlaghzadeh et al., 2021[6]). A recent prospective study in India found advanced liver disease to account for 2.7 % of cases (Sulaiman et al., 2022[8]), while another study reported hypercalcemia in 8.3 % of patients with chronic liver disease (Vijayan et al., 2023[10]). Symptoms of hypercalcemia are rare but can include confusion, weakness, constipation, polyuria, and atrioventricular block. Herein, we discuss one such case of hypercalcemia related to end-stage liver disease. 

A 49-year-old female with decompensated alcohol-related cirrhosis with previous Transjugular intrahepatic portosystemic shunt (TIPS) procedure and chronic kidney disease secondary to IgA Nephropathy, presented with altered mental status, inadequate dietary intake, and fatigue. Vitals were stable and general examination revealed dry and icteric mucous membranes, generalized weakness, and slow verbal responses. Neurological examination showed mild asterixis with Grade I encephalopathy. The laboratory findings included ammonia level >200 micromol/L (10-40 micromol/L), bilirubin 2.6 mg/dL, creatinine 2.0 mg/dL, and notably, elevated corrected calcium levels at 14.0 mg/dL. Pertinent negatives included the absence of fever, ascites, and leukocytosis. The patient was treated with intravenous fluids, lactulose, rifaximin, and elemental zinc for encephalopathy, acute kidney injury, and hypercalcemia. Workup of hypercalcemia was unremarkable including serum PTH, PTHrP, Thyroid stimulating hormone (TSH), Alpha-fetoprotein (AFP), Angiotensin converting enzyme (ACE), Vitamin A, Vitamin D (25-hydroxyvitamin D and 1,25-dihydroxyvitamin D) levels, and Serum Protein Electrophoresis (SPEP). The results of lab investigations and workup are shown in Supplementary information, Table 1. Skeletal survey, CT abdomen and pelvis were unremarkable. Following an extensive negative workup, a diagnosis of idiopathic hypercalcemia secondary to end-stage liver disease was made. Calcitonin therapy was given for two days with minimal improvement. Zoledronic acid led to normalization of calcium level by day 7 of administration.

Liver disease is an uncommon and poorly recognized cause of hypercalcemia. Hypercalcemia due to end-stage liver disease remains a diagnosis of exclusion and responds well to bisphosphonate treatment (Kuchay et al., 2016[4]; Ragate et al., 2021[7]). The pathogenesis of hypercalcemia in chronic liver disease is not fully understood, but there are speculations about heightened inflammation leading to increased bone resorption (Athonvarangkul et al., 2020[1]; Cadranel et al., 1989[2]). In contrast to our case, existing literature reveals rapid improvement (<3 days) with bisphosphonates and calcitonin (Ragate et al., 2021[7]; Jadoon et al., 2021[3]). Notably, the transient nature of this hypercalcemia also stands out as an attribute worth highlighting. Hypercalcemia in Chronic liver disease (CLD) correlates with the severity of liver disease and bilirubin levels (Vijayan et al., 2023[10]). Also, the duration of hypercalcemia was positively associated with 90-day mortality in these patients (Majety et al., 2022[5]). Although hypercalcemia is a rare and uncommon encountered electrolyte abnormality in patients with chronic liver disease (CLD), It is commonly observed in the inpatient setting. Therefore with the rising prevalence of chronic liver disease, clinicians should consider advanced liver disease as a potential cause.

## Conflict of interest

The authors have no conflict of interest to declare.

## Supplementary Material

Supplementary information
