# Supplementary material for: Unveiling the enigma: a case of hypercalcemia in end-stage liver disease
Source: EXCLI J. 2025 Aug 18;24:1027–8. doi: 10.17179/excli2025-8663 (PMC12635825; doi:10.17179/excli2025-8663)
Supplement: Supplementary information [file EXCLI-24-1027-s-001.pdf]

EXCLI Journal 2025;24:1027-1028 – ISSN 1611-2156
Received: June 19, 2025, accepted: June 28, 2025, published: August 18, 2025

Supplementary information to:

Letter to the editor:

UNVEILING THE ENIGMA: A CASE OF HYPERCALCEMIA IN END-STAGE LIVER DISEASE

Rutvikkumar Jadvani, Abul Hasan Shadali Abdul Khader, Meenu Singh*

Division of General Internal Medicine, University of Utah School of Medicine, Salt Lake City, Utah, USA

* **Corresponding author:** Meenu Singh, MD, Division of General Internal Medicine, University of Utah School of Medicine, 30 North Mario Capecchi Dr, North Salt Lake City, UT 84112. Phone: 801-581-7822 Fax: 801-585-9166,
E-mail: meenu.singh@hsc.utah.edu

https://dx.doi.org/10.17179/excli2025-8663

This is an Open Access article distributed under the terms of the Creative Commons Attribution License (https://creativecommons.org/licenses/by/4.0/).

Table 1: Lab investigations and workup

Lab test	Lab result
**Hemoglobin**<br>**Total leukocyte count**<br>**Platelet count**	12.4 g/dl<br>8100 /mm^3<br>1,60,000 /mm^3
**Corrected calcium**<br>**Phosphorus**	14.0 mg/dL<br>3.3 mg/dL
**Creatinine**<br>**Urinary calcium**	2.0 mg/dL<br>211 mg/day
**Albumin**<br>**Globulin**<br>**Bilirubin**<br>**Ammonia level**	2.6 g/dL<br>2.0 g/dL<br>2.6 mg/dL<br>>200 micromol/L
**25-OH Vitamin D**<br>**1,25-OH Vitamin D**<br>**Serum PTH**<br>**PTHrP**<br>**TSH**<br>**AFP**<br>**ACE**<br>**Vitamin A**	33 ng/mL<br>23.4 pg/mL<br><5 pg/mL<br><2.5 pmol/L<br>3.9 mIU/L<br>3.2 IU/L<br>22 U/L<br>0.07 µmol/L
**Cortisol**	13 mcg/dL
**CEA**<br>**CA 19-9**	1.3 ng/mL<br>22 U/mL

PTH - Parathyroid hormone; PTHrP - Parathyroid hormone-related peptide; TSH - Thyroid stimulating hormone; AFP - alpha-fetoprotein; ACE - Angiotensin-converting enzyme; CEA - carcinoembryonic antigen
